# Neighborhood Conditions and Type 2 Diabetes Risk among Latino Adolescents with Obesity in Phoenix

**DOI:** 10.3390/ijerph19137920

**Published:** 2022-06-28

**Authors:** Connor M. Sheehan, Esther E. Gotlieb, Stephanie L. Ayers, Daoqin Tong, Sabrina Oesterle, Sonia Vega-López, Wendy Wolfersteig, Dulce María Ruelas, Gabriel Q. Shaibi

**Affiliations:** 1 School of Social and Family Dynamics, Arizona State University, Tempe, AZ 85281, USA; 2 Southwest Interdisciplinary Research Center, School of Social Work, Arizona State University, Phoenix, AZ 85004, USA; esther.gotlieb@asu.edu (E.E.G.); stephanie.l.ayers@asu.edu (S.L.A.); sabrina.oesterle@asu.edu (S.O.); 3 School of Geographical Sciences & Urban Planning, Arizona State University, Tempe, AZ 85281, USA; daoqin.tong@asu.edu; 4 College of Health Solutions and Southwest Interdisciplinary Research Center, Arizona State University, Phoenix, AZ 85004, USA; sonia.vega.lopez@asu.edu; 5 School of Social Work, Arizona State University, Tempe, AZ 85281, USA; wendy.wolfersteig@asu.edu; 6 College of Nursing & Healthcare Professions, Grand Canyon University, Phoenix, AZ 85017, USA; dulce.ruelas@gcu.edu; 7 Center for Health Promotion and Disease Prevention, Edson College of Nursing and Health Innovation, Arizona State University, Phoenix, AZ 85004, USA; gabriel.shaibi@asu.edu

**Keywords:** diabetes, neighborhoods, adolescence, obesity, Latinos

## Abstract

Type 2 Diabetes (T2D) has reached epidemic levels among the pediatric population. Furthermore, disparities in T2D among youth are distributed in a manner that reflects the social inequality between population sub-groups. Here, we investigated the neighborhood determinants of T2D risk among a sample of Latino adolescents with obesity residing in Phoenix, Arizona (*n* = 133). In doing so we linked together four separate contextual data sources: the American Community Survey, the United States Department of Agriculture Food Access Research Atlas, the Arizona Healthy Community Map, and the National Neighborhood Data Archive to systematically analyze how and which neighborhood characteristics were associated with T2D risk factors as measured by fasting and 2-h glucose following a 75 g oral glucose tolerance test. Using linear regression models with and without individual/household covariates, we investigated how twenty-two housing and transportation sociodemographic and built and food environment characteristics were independently and jointly associated with T2D risk. The main finding from these analyses was the strong association between the density of fast food restaurants and 2-h glucose values (b = 2.42, *p* < 0.01). This association was independent of individual, household, and other neighborhood characteristics. Our results contribute to an increasingly robust literature demonstrating the deleterious influence of the neighborhood food environment, especially fast food, for T2D risk among Latino youth.

## 1. Introduction

Type 2 diabetes (T2D) has reached near epidemic levels among the pediatric population in the United States [1,2]. Recent estimates suggest that more than 2% of children have been diagnosed with diabetes; a rate that has nearly doubled since the turn of the 21st century [3]. Diabetes has profound and lifelong implications, increasing the risk of subsequent chronic conditions such as cardiovascular disease [4], and is strongly associated with premature mortality [5]. Diabetes also requires intensive and consistent management [6], is financially expensive [7], and is associated with other non-health related outcomes such as reduced educational attainment [8], as well as increased personal stress and stress among family members [9].

Concerningly, T2D remains distributed in a manner that reflects the social inequality among the pediatric population. Latino adolescents, in particular, have a significantly higher incidence and prevalence of T2D, especially when compared to non-Latino White children [10]. While extensive research has focused on the individual determinants of T2D among youth generally and among Latinos specifically, T2D among Latino youth has continued to climb [11,12,13]. Despite this increasing and disproportionate increase, relatively less research has examined how contextual factors, such as neighborhoods, are associated with T2D risk specifically among Latino youth (notable exceptions discussed below). Yet these contextual factors in general and neighborhood factors in particular could be critical to the broader understanding of T2D disparities among Latino youth.

Indeed, the relative inattention to the importance of neighborhood determinants of T2D among Latino youth is notable given that Latino youth systematically and increasingly reside in low-resourced and segregated neighborhoods [14,15,16]. Low resourced and segregated neighborhoods have been shown to contribute to T2D risk among adults [17]. These neighborhoods are shaped by historical, environmental, demographic, social, and economic processes [14,15,18,19,20] that influence health and disease [21,22], including the risk of T2D [23,24]. However, the influence of these specific neighborhood features may uniquely vary markedly in relation to T2D risk among Latino adolescents. Therefore, understanding the influence of specific neighborhood characteristics for Latino adolescents is critical to understanding the “upstream” and contextual determinants of T2D that shape health behaviors to influence T2D risk factors including obesity and insulin resistance [25,26]. Understanding which specific neighborhood characteristics are most important for diabetes risk may also help in the development of tailored contextual interventions.

To further elucidate the importance of specific neighborhood conditions for diabetes among Latino adolescents, we combined four neighborhood data sources to systematically analyze how and which housing, transportation, demographic, socioeconomic, and the built/food environment factors were associated with proximal markers of T2D among a sample of socioeconomically low resourced Latino youth with obesity in the greater Phoenix metropolitan area of Arizona. In doing so, we carefully adjusted for important individual and household level risk factors as well as other neighborhood characteristics and worked with a Community Advisory Board (CAB) to receive feedback regarding our approach as well as the implications of our findings. This research design allowed us to identify potentially modifiable aspects of neighborhoods that may stem the higher rates of T2D among Latino youth. Overall, we aim to document which specific neighborhood factors predict objectively measured diabetes risk adjusting for individual, household, and other neighborhood characteristics among one of the fastest growing and highest T2D risk segments of the American population: Latino youth in one of Americas largest and most rapidly growing urban environments: Phoenix, Arizona.

### Literature Review

Spatial contexts, such as neighborhoods, condition many individual behaviors and exposures that contribute to T2D risk. For instance, neighborhoods shape access to healthy and unhealthy foods [27], affect the ability and propensity to be physically active [28], mediate exposure to environmental pollutants (e.g., air [29] and noise pollution [30]), influence housing conditions [31], structure socioeconomic opportunities and trajectories [32], impact free time [33], determine schools and school funding levels [34,35], drive sleep patterns [36], and can also cause stress or buffer stressors through factors such as community cohesion [37].

Accordingly, research regarding the neighborhood determinants of health and diabetes has grown precipitously [38], yet much of this research has compared socioeconomically advantaged neighborhoods to socioeconomically disadvantaged neighborhoods [17,36,39,40,41]. These research designs have elucidated the critical, even causal, importance of neighborhood socioeconomic conditions for health outcomes, including diabetes [17]. However, research designs that compare high resource versus low resource neighborhoods risk implicitly assuming that more proximal neighborhood determinants of health across neighborhoods (e.g., access to food) are similarly associated with health outcomes in advantaged and disadvantaged neighborhoods. However, these relationships likely vary dramatically across types of neighborhoods and increasingly scientists have stressed the importance of within-group analyses in addition to between-group analyses for understanding disparities [42].

For instance, past research has found that the presence of fast food restaurants increases the risk of negative health outcomes like diabetes [38], but this relationship likely varies not only between more and less advantaged neighborhoods, but also within neighborhoods. More advantaged neighborhoods likely have other healthy food options readily available that may make fast food less alluring [43], and low resourced neighborhoods may not have other food options which may exacerbate their influence on diabetes [44]. Overall, even as researchers have increasingly documented that socioeconomically disadvantaged neighborhoods can negatively influence health and diabetes, we contribute to this research by analyzing multiple neighborhood characteristics within predominantly low-resourced neighborhoods with high concentrations of Latino residents to further elucidate which characteristics, and their variation within disadvantaged neighborhoods, are most important for T2D risk.

Despite Latino children and adolescents’ disproportionate share of T2D cases [1], there is also comparably little research regarding the importance of neighborhood characteristics focusing explicitly on the Latino pediatric population in general and Latino adolescents in particular. This is an important oversight, as one study indicated that Latino youth’s diabetes risk was especially sensitive to neighborhood socioeconomic conditions compared to youth of other racial/ethnic backgrounds in Chicago [45]. Other research regarding neighborhood determinants of diabetes among Latino youth has utilized focus groups and surveys in Northern California and stressed that the manner in which neighborhoods can structure food availability is critically important for diabetes risk [46]. They found that food in Latino neighborhoods is often unhealthy and inexpensive, while healthy food is often unavailable [46]. Similarly, a study in Southern California concluded that fast food density was associated with higher insulin resistance, while park space was associated with lower insulin resistance among Latino children who were overweight or obese [26]. A conceptual overview also focused on the mechanisms through which ethnic enclaves may increase diabetes risk among Latinos, noting the specific importance of how neighborhoods may determine access to food among Latinos [47]. Taken together, these past studies stress the importance of socioeconomic and spatial/built environmental processes as well as the food environment in particular for shaping T2D risk among Latinos.

We build on this research by including multiple measures of the neighborhood food environment as well as sociodemographic, built environment, housing, and transportation neighborhood characteristics and examine the independent and collective associations between multiple neighborhood characteristics from multiple neighborhood datasets in predicting T2D risk in the greater Phoenix metropolitan area. With this approach, we aim to provide a comprehensive documentation of which neighborhood characteristics are important for Latino adolescents’ T2D risk. Given the findings of past research [45,46], we anticipate that the neighborhood fast food environment will be especially important for shaping the T2D risk of Latino adolescents even after adjusting for individual and household characteristics as well as neighborhood sociodemographic, built environment, housing, and transportation characteristics.

## 2. Materials and Methods

### 2.1. Data

To understand how neighborhood characteristics are associated with diabetes risk among Latino youth, the present study linked individual-level data about diabetes risk collected from Latino youth in the greater Phoenix metropolitan area to neighborhood characteristics (discussed below) based on census tracts identified by participants home addresses, and all respondents were successfully georeferenced to four neighborhood datasets (discussed below).

#### 2.1.1. Participant-Level Data

Individual respondents (*n* = 133) were recruited through a network of schools, community centers, and healthcare organizations in Phoenix, Arizona as part of a culturally-grounded lifestyle intervention aimed at reducing T2D risk among Latino adolescents. The design of the study and effects of the intervention have been described elsewhere [48,49]. Pre-intervention data were collected between 2013–2015 and used for the current analysis. The project was approved by the Institutional Review Board at Arizona State University (ASU), and all participants and a parent/legal guardian provided written informed assent and consent prior to data collection. Participants met the following inclusion criteria: (1) self-identification as Latino, (2) age 14–16, and (3) met criteria for obesity (defined as a BMI ≥ 95th percentile for age and sex or a BMI ≥ 30 kg/m^2^). Participants were excluded if they were (1) taking medication(s) or diagnosed with a condition that influences carbohydrate metabolism, physical activity, or cognition; (2) diagnosed with T2D; (3) currently enrolled (or within previous six months) in a formal weight loss program; or (4) were diagnosed with depression or any other condition that may impact quality of life. Participant’s home addresses were georeferenced to be able to link them to census tract-level data about neighborhoods. The sample was geographically dispersed throughout Phoenix, but overwhelmingly individuals lived in low-resourced neighborhoods (discussed further below).

#### 2.1.2. Neighborhood-Level Data

Four unique sources of neighborhood data were merged to create a more comprehensive dataset of neighborhood characteristics across the greater Phoenix metropolitan area, which includes the cities of Avondale, Chandler, Fountain Hills, Gilbert, Glendale, Phoenix, Scottsdale, and Tempe. These datasets include the American Community Survey [50], the United States Department of Agriculture Food Access Research Atlas [51], the Arizona Healthy Community Map [52], and the National Neighborhood Data Archive [53]. In the interests of parsimony and transparency, we discuss each in greater depth in the Supplemental Materials.

### 2.2. Measures

#### 2.2.1. Household and Individual Measures

Participants arrived at the ASU Clinical Research Unit at ~8:00 a.m. after an overnight fast for assessment of adiposity and T2D risk. Height and weight were assessed to the nearest 0.1 cm and 0.1 kg to determine BMI and BMI percentiles (BMI%). T2D risk was determined by a 2-h oral glucose tolerance test (OGTT). Blood samples were collected before and 2-h after ingestion of 75 g of glucose in solution by trained research nurses. Samples were used to assess fasting and 2-h glucose concentrations by the cobas c111 analyzer from Roche Diagnostics (Indianapolis, IN, USA). Abnormal OGTT results were determined by the American Diabetes Association criteria for prediabetes with either a fasting glucose value ≥100 mg/dL and/or a 2-h glucose value ≥140 mg/dL. Participants meeting American Diabetes Association criteria for T2D (fasting glucose value ≥126 mg/dL or a 2-h glucose value ≥200 mg/dL) were excluded from the study and referred for follow-up care. In addition to T2D risk, analyses accounted for participant age and sex, parent/guardian-reported parental education (less than high school (reference), high school, and more than a high school education), household size, and parent/guardian-reported monthly household income (in dollars) to document the influence of neighborhood factors above and beyond individual/household characteristics.

#### 2.2.2. Neighborhood Measures

We measured neighborhood characteristics in three categories: housing and transportation, sociodemographic, and built and food environment, all at the tract-level. The source of each of these measures is provided in Table 1, with additional details regarding the datasets in the Supplemental Materials. In terms of the housing and transportation characteristics, we included the average household size, percentage of residents living in the same house in the past year, the percentile of households who have lived in the neighborhood over the past seven years, the percentage of housing units with no vehicle, and the percentage of rental units that are vacant.

In terms of neighborhood sociodemographic characteristics we included the gross median monthly rent (scaled in $100 dollars), low-income tract status designation (indicated by at least 20% of households being impoverished), the percentage of residents who lived below the federal poverty line, the unemployment percentage, the percentage of residents who were college graduates, and the percentage of residents who self-identified as Hispanic/Latino. We also included two composite indices. The first measure, concentrated neighborhood disadvantage, is a composite index of poverty consistent with the previous research [53,54]. This index was comprised of: (1) the percentage of individuals below the poverty line; (2) the percentage of individuals on public assistance; (3) the percentage of female-headed households; (4) the percentage unemployed; and (5) the percentage under 18 years of age. These percentiles were z-score transformed (i.e., standardized across Maricopa County, Arizona, where the Phoenix metro area is located) and then averaged into an index to represent the extent of deprivation. Consistent with previous research [53,54], cut-offs were then applied to these indices at the 75th percentile to identify areas of high concentrated disadvantage. The second composite index, from the Arizona Healthy Community Map, measures tract health based on 12 domains such as access to care, affordable quality housing, community safety, healthy community design, social and cultural cohesion, and social justice. More detailed information regarding the Arizona Healthy Community Map composite including the indicators used in its construction is provided in the Supplemental Materials and is also available elsewhere [51].

Built environment characteristics included the percentage of street light brightness (nW/cm^2^ sr^−1^), the percentile of traffic volume/proximity to traffic, the percentile of public park space within one-square mile of the Census tract, the walkability score percentile [51], the percentile of lack of supermarkets (i.e., “food deserts”) [51], the number of full-service restaurants per square mile, the number of fast food restaurants per square mile, the number of alcoholic drinking places per square mile (i.e., bars), and the number of snack shops per square mile.

### 2.3. Methods

We first calculated the neighborhood and individual/household descriptive statistics for the sample census tracts and respondents, respectively. We compared the neighborhood characteristics relative to the aggregated greater Phoenix metropolitan area for all neighborhood characteristics and presented them in Table 1. We calculated T-tests and the proportion T-tests where appropriate to formally test if the tracts where the respondents reside significantly differed from the broader Phoenix area for each neighborhood characteristic.

Next, we fitted a series of ordinary least squares (OLS) regression models using neighborhood characteristics to predict fasting and 2-h glucose levels. In the first set of models, we fitted an individual model for every neighborhood covariate with no other covariates in the model in order to provide a bivariate association between the neighborhood measure and glucose value. Next, in the second model, we added the individual/household covariates to each individual model to examine if controlling for the individual/household characteristics reduced the associations between each neighborhood characteristic and T2D risk. In the third set of models, we were interested in understanding how controlling for the neighborhood indicators within each domain influenced the associations. That is, in Model 3, we fitted three separate models where we separately added all neighborhood covariates within the different types of neighborhood measures (i.e., all housing/transportation neighborhood measures in one model, all sociodemographic neighborhood measures in one model, and all built/food environment neighborhood measures in one model) accounting for all of the individual and household characteristics. Finally, we fitted a full model with all of the individual and neighborhood covariates into one model. This allowed us to examine the influence of neighborhood characteristics net of individual characteristics without other neighborhood characteristics and also net of numerous other neighborhood characteristics. For the joint models, variance inflation factor (VIF) tests indicated little issues with multicollinearity. All regression models were estimated using Stata 14.2. [55] The sample population resided across a total of 92 unique Census tracts. Accordingly, all standard error estimates were adjusted for clustering at the Census tract level. We also conducted additional analyses and found little differences across gender.

## 3. Results

**Figure 1:**
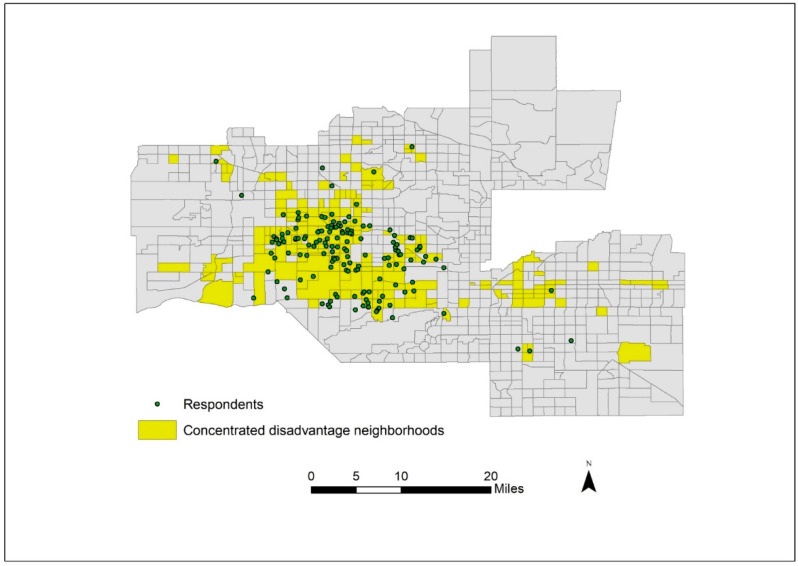
Distribution of Sample and Concentrated Disadvantaged in the Phoenix Chandler Area.

The sample was characterized by a high degree of risk factors for T2D including obesity (BMI percentile = 98.1 ± 1.4), and 15% meeting the American Diabetes Association criteria for prediabetes. Table 1 presents the individual and neighborhood level descriptive statistics for the analytic sample (133 youth living in 92 unique Census tracts). Just over half of the sample was female; 41% were 14 years old, 34% were 15 years old, and 25% were 16 years old. Over half of the respondent’s parents did not have a high school education (63.2%), and the modal level of monthly household income was between $1000 and $2000 dollars. The neighborhood measures are compared to the Phoenix metropolitan area. Overall, the respondents resided in neighborhoods with significantly lower levels of resources compared to the Phoenix metropolitan area. For instance, almost 15% of residents in sample neighborhoods did not own a vehicle, which was nearly double that of the greater Phoenix area (7.6%, a statistically significant (*p* < 0.05) difference). Respondents’ Census tracts were primarily low-income tracts (93.2%) with poverty rates over 40%, a Census indicator of “high poverty neighborhoods” [36], and this was more than double that of the Phoenix area (42.2%, a statistically significant (*p* < 0.05) difference). Similarly, 84.2% of the tracts in the analytic sample were in areas of high concentrated disadvantage compared to 27.0% of tracts in the Phoenix area (a statistically significant (*p* < 0.05) difference). Figure 1 shows the degree of spatial dispersion of the sample and that the sample also overwhelmingly resides in tracts characterized by concentrated disadvantage.

The only neighborhood characteristic that was significantly associated with fasting glucose levels was traffic volume/proximity (b = −0.08, *p* < 0.05). Given the lack of significant associations with fasting glucose, we did not pursue additional models for this T2D risk indicator. Table 2 presents coefficients from linear regression models predicting 2-h glucose among Latino youth. The first set of models (Model 1) provides the bivariate association between each neighborhood characteristic and 2-h glucose values. In terms of neighborhood housing/transportation characteristics, the higher the percentage living in the same house in last year was associated with significantly lower 2-h glucose values (b = −0.65, *p* < 0.05), while the percent of rental vacancy was associated with higher 2-h glucose (b = 0.66, *p* < 0.05). That is, residential instability was significantly associated with 2-h glucose values. None of the neighborhood sociodemographic measures were meaningfully associated with 2-h glucose values, which is not surprising given that the sample is relatively homogenous in terms of neighborhood socioeconomic status. For the built environment, traffic was negatively associated with 2-h glucose values (i.e., more traffic was associated with lower 2-h glucose values; −0.38, *p* < 0.01), while the number of fast food restaurants was positively associated with 2-h glucose values (b = 2.42, *p* < 0.01), as each additional fast food restaurant per square mile was associated with a 2.42 mg/dL increase in 2-h glucose, which is quite a strong relationship given the range of 0–9 of fast food restaurants per square mile in each tract and a standard deviation of 2.0.

The second set of models (Models 2) was a series of models where each neighborhood characteristic was modeled separately to predict 2-h glucose value with individual level covariates included. Despite the inclusion of individual/household covariates, the substantive results did not change dramatically. Indeed, in these models one-year housing stability was once again negatively associated with 2-h glucose value (b = −0.83, *p* < 0.01), while no vehicle percentage (b = 0.50, *p* < 0.05) and rental vacancy (b = 0.81, *p* < 0.01) were positively associated with 2-h glucose values. In terms of neighborhood socioeconomic characteristics, gross median rent was negatively associated with 2-h glucose values (b = −2.65, *p* < 0.05). For the built/food environment characteristics, street light brightness had a positive association with 2-h glucose value (b = 0.32, *p* < 0.05), traffic proximity had a negative association (b = −0.43, *p* < 0.01), and fast food density continued to have a significant and strong positive association (b = 3.03, *p* < 0.01).

Table 3 provides results from Model 3 and Model 4. Model 3 adjusted for all neighborhood indicators within each domain, as well as including the individual/household covariates. In these models, none of the housing/transportation or neighborhood sociodemographic characteristics were statistically significant. Notably, the associations for living in the same house and rental vacancies only slightly declined, while the confidence intervals widened, suggesting that the association may be robust to adjusting for other factors, but we were unable to estimate them with precision given the small sample. However, traffic proximity (b = −0.32, *p* < 0.05) and fast food density (b = 3.34, *p* < 0.05) remained statistically significant predictors of 2-h glucose values.

Finally, in Model 4, we fitted a joint model where all neighborhood characteristics were included in one model that also accounted for the individual and household characteristics. Many of the neighborhood associations were no longer statistically significant. Indeed, when neighborhood housing/transportation, socioeconomic, and built environment factors were included in the model, the neighborhood food environment measures remained statistically significant. Specifically, fast food restaurants per square mile was independently associated with higher 2-h glucose values (b = 5.48, *p* < 0.001), while full-service restaurants were negatively associated with 2-h glucose values (b = −2.00, *p* < 0.01). Also worth noting is that the percentile of park area was negatively associated with 2-h glucose values in the fully adjusted model at the marginal level (b = −0.28, *p* < 0.10).

## 4. Conclusions

Despite substantial advances in the understanding of the biological and behavioral determinants of T2D risk in youth, how social and structural factors contribute to T2D disparities among high-risk pediatric sub-groups remains relatively less well understood [1,2]. The increasing T2D rates among Latino youth [3] suggest that a more comprehensive approach that considers contextual factors is warranted in this growing segment of the pediatric population. Thus, we analyzed the associations between a wide array of neighborhood characteristics and T2D risk, assessed by an oral glucose tolerance test, among a sample of low-income, Latino adolescents with obesity, living in low resourced neighborhoods in Phoenix, Arizona. We contributed to the existing research in the following three ways.

First, rather than using one data source, we merged together four unique neighborhood data sources to create one of the most comprehensive datasets of neighborhood characteristics in predicting diabetes among Latino adolescents heretofore analyzed. Despite the diverse and extensive measures of neighborhood characteristics, our substantive results largely replicated the findings of past researchers in Northern [46] and Southern [26] California which highlighted the importance of the neighborhood food environment, especially fast food availability, in shaping T2D risk among Latino children. Specifically, even in models that included individual and household characteristics as well as a wider range of other neighborhood characteristics, including neighborhood socioeconomic status and other measures of the food and built environment, we found Latino youth who lived in neighborhoods with a greater number of fast food restaurants had higher levels of T2D risk, as each additional fast food restaurant per square mile in the tract increased 2-h glucose levels by 3.03 md/dL in the full model. Thus, our study adds to the mounting evidence [26,46,47] stressing the deleterious influence of fast food restaurants on T2D risk for Latino youth. These increasingly robust findings stress that the fast food environment could be a potentially modifiable way to stem the disproportionate levels of T2D among Latino youth.

In more detail, we found that the fast food density was the most consistently strong predictor of 2-h glucose values, and that it remained statistically significant even after adjusting for individual, household, and other neighborhood characteristics. In contrast, we also found that full “sit down” restaurants were negatively associated with diabetes risk in the fully adjusted model. While it is less clear why full “sit down” restaurants were inversely associated with T2D risk, the density of fast food may expedite the dietary acculturation process that has been linked to higher risk of T2D among Latinos [25,56]. That is, when Americanized fast food is the only or most densely food available, dietary acculturation could be more forced by proximity (there are little other options for food). In contrast, more traditional or healthier options may be available at sit down restaurants. Together, these results stress the importance of the neighborhood food environment, especially fast food restaurants, for shaping diabetes risk among Latino youth with obesity. Future research with more detailed dietary and behavior information among Latino children could further elucidate why the fast food environment of neighborhoods may be uniquely consequential for diabetes risk among Latino children.

The strength of these findings in conjunction with previous research on this topic [26,46], lends support to policy-based approaches for preventing T2D [57]. Although consensus on the types of and efficacy for policies to improve obesity-related outcomes is lacking [58], recent models suggest that taxing unhealthy foods while subsidizing fruits and vegetables may lead to health gains and cost savings at the population level [59]. Extending this concept to the current findings, one could imagine that taxing fast food to subsidize community gardens (see discussion below) could decrease diabetes disparities among Latino adolescents. From a structural perspective, changes to the built environment within low resourced communities could further support health promotion and diabetes prevention behaviors [60].

A second contribution to the current literature is our exclusive focus on a sample of Latino adolescents with obesity living in primarily low resourced neighborhoods. This is in contrast to previous neighborhood determinants of health research [36,39], which has tended to compare advantaged to disadvantaged neighborhoods or the influence of neighborhood characteristics across race/ethnic groups [45]. The direct and indirect mechanisms through which neighborhoods influence health likely vary across levels of neighborhood advantage and among Latinos, in particular, who tend to live in specific neighborhoods/ethnic enclaves [47] that may uniquely shape their food consumption, health, and diabetes risk. By focusing exclusively on Latino youth in low resourced neighborhoods, we were able to understand specific neighborhood factors relevant to their diabetes risk; indeed, we found that the food environment and specifically fast food density was the strongest neighborhood predictor of diabetes. These association may not be apparent among more advantaged segments of the population who have more access to healthy food or among other racial/ethnic populations.

A third advancement was focusing on Latino youth who are at risk for T2D due to elevated adiposity [61]. Youth with obesity may be especially susceptible to neighborhood characteristics [62] given how neighborhoods can structure the food environment, as well as opportunities for physical activity. While we found that restaurants, especially fast food restaurants, were significant predictors of 2-h glucose concentrations, replicating past research focusing on children with overweight and obesity [26]. Taken together, these results indicate that the contextual processes which shape obesity risk may also shape diabetes risk.

Despite the contributions to the literature, we acknowledge that there are important limitations to consider. First, most neighborhood research has struggled with issues related to selection (among other issues) that prevents causal inference. That is, the certain amenities that may attract people to certain neighborhoods may influence their health. However, adolescents have comparably less agency in deciding their neighborhoods than adults, which could minimize this limitation, and previous causal designs [17] have also stressed the importance of neighborhood for diabetes risk. Second, our research is cross-sectional, which inhibits our understanding regarding how exposure to these specific neighborhood characteristics influences diabetes risk. Future research with longitudinal measures could better document how gradual exposure to neighborhood characteristics may influence diabetes risk. Third, we only analyzed Latino youth with obesity in the Phoenix metro area, and this limits the generalizability of our results to other areas in the country. However, we are reassured as our substantive results were similar to those in other areas [26,46]. Still, the extent to which our results are generalizable to other racial/ethnic adolescents or Latino youth who do not have obesity remains unclear. Latinos may be particularly sensitive to neighborhood factors in general (as shown in previous research [45]) and to fast food in particular (given dietary acculturation patterns [25,56]). Future research should consider analyzing the neighborhood determinants of other populations to better understand if the patterns we document are unique. That is, other neighborhood characteristics may be more or less important for conditioning diabetes risk among other populations. While we investigated a relatively comprehensive list of neighborhood characteristics, it is not all encompassing (nor could it be). Thus, future research should continue to investigate how neighborhood characteristics from multiple sources of data are associated with diabetes risk. Fourth, the neighborhood characteristics were not always from the same year as the respondent’s survey. However, the discrepancies were often minimal and while neighborhoods change, the change is often gradual [39]. Census tracts may not perfectly reflect a neighborhood nor individuals’ activity spaces within neighborhoods, but previous research has found that they are a generally reliable way to encompass neighborhoods [63]. Indeed, Census tracts are a ubiquitously used method of classifying neighborhoods [17,64] as they are designed to characterize neighborhoods, are created to reflect the sociodemographic profile of an area, and are based on physical features [65]. Additionally, census tracts have real strengths, specifically that many neighborhood databases and characteristics are specified and measured at the tract level, allowing researchers to merge multiple sources of information regarding neighborhood characteristics, and utilizing census tracts allows comparability with other research.

We discussed our research and findings with a Community Advisory Board (CAB), that represents stakeholders from various sectors and organizations across Phoenix, Arizona. CAB members facilitate communication between researchers and community-based agencies with the goal of enhancing the potential impact of research to reduce health disparities in the local community. After presenting the findings described above, the CAB identified additional limitations as well as opportunities for consideration. For instance, they noted that while we accounted for household characteristics, we did not include measures of multigenerational households, which could influence diabetes risk or buffer/amplify neighborhood characteristics (unfortunately these measures were unavailable in our dataset). They also noted the importance of real-time tracking to better account for neighborhood exposures and representation which may be critical in highly mobile communities such as those experiencing unstable housing or frequent immigration. CAB members expressed that neighborhood-level research has the potential to exacerbate discrimination, targeting, and/or racial profiling in communities of color, and that this may be particularly relevant with the recent attention on the social determinants of health and health disparities.

CAB members also noted that neighborhoods are nuanced, and that relying solely on census tract level data to describe neighborhoods may be limiting. In the context of mobility, researchers would benefit from appreciating that some individuals may belong to multiple neighborhoods, and specific to diabetes, they should consider how changes in the continuity of resources may affect health. While our findings indicated the deleterious aspects of neighborhood characteristics on diabetes risk factors, CAB members noted that there may be strengths within neighborhoods, and that these strengths can positively influence community health. As an example, contrary to our findings on the relationship between fast food density and T2D risk, the CAB pointed out that many neighborhoods have robust community gardens to support access to fresh fruits and vegetables. Creative solutions may be needed to identify community gardens as well as other neighborhood level assets that may not otherwise be apparent to researchers using only large, publicly available datasets [66]. Overall, engaging with CABs allows researchers to ground their work in the local community context and supports shared decision making, identifies the limitations more clearly, and enhances the potential impact of research within the local community [67].

## Figures and Tables

**Table 1:** Descriptive Statistics, Latino Youth in Phoenix with Obesity (*n* = 133), 2013–2015.

	Individual/Household Characteristics (*n* = 133)									
	Mean or %	SD	Min	-	Max					
**Glucose Concentrations**										
Fasting Glucose	92.9	6.4	79	-	112.8					
2-h Glucose	129	25.1	62.5	-	198					
**Respondent**										
*Gender*										
Male (%)	46.6									
Female (%)	53.4									
*Age*										
14 (%)	41.4									
15 (%)	33.8									
16 (%)	24.8									
**Parental Characteristics**										
*Parental Education*										
No high school diploma (%)	63.2									
High school diploma (%)	21.8									
Some college/College graduate (%)	15									
**Household Characteristics**										
*Household Income Per Month*										
$0–$1000 (%)	28.6									
$1001–$2000 (%)	46.6									
>$2000 (%)	24.8									
Household Size	5.1	1.5	2	**-**	11					
	**Neighborhood-Level Characteristics**									
	***Analytic Sample Neighborhood Characteristics (92 Tracts)***					***Phoenix/Chandler Neighborhood Characteristics***				
	**Mean or %**	**SD**	**Min**	**-**	**Max**	**Mean or %**	**SD**	**Min**	**-**	**Max**
**Neighborhood Housing and Transportation**										
**Average Household Size (# of persons) ^a^**	**3.5**	**0.6**	**1.9**	**-**	**4.8**	**2.8**	**0.6**	**1.3**	**-**	**5**
Living in Same House 1-year (%) ^a^	**79.7**	**7.9**	**56.1**	**-**	**94.6**	**80.1**	**9.4**	**32.4**	**-**	**100**
**Living in Same Neighborhood 7 years (%) ^c^**	**28.1**	**19.2**	**0.9**	**-**	**82.4**	**42.1**	**27.6**	**0.1**	**-**	**99.6**
**No Vehicle (%) ^b^**	**14.6**	**10.6**	**0**	**-**	**39.5**	**7.6**	**8.4**	**0**	**-**	**53.5**
Rental vacancy (%) ^a^	9.8	6.7	0	**-**	29.2	8.4	7.7	0	-	43.5
**Neighborhood Sociodemographic Characteristics**										
**Gross Median Rent ($) ^a^**	**854.7**	**214.4**	**331**	**-**	**1597**	**1059.9**	**303.1**	**373**	**-**	**2000**
**Low Income Tract (% Yes) ^b^**	**93.2**	**25.2**	**0**	**-**	**1**	**42.2**	**49.4**	**0**	**-**	**100**
**Poverty Rate (%) ^a^**	**40.6**	**14.2**	**9.2**	**-**	**72.3**	**18.6**	**15.5**	**0**	**-**	**100**
**Unemployment (%) ^c^**	**37.7**	**22.2**	**2.3**	**-**	**90.4**	**55.8**	**27.1**	**0.1**	**-**	**100**
**College graduates (%) ^a^**	**9.7**	**7.9**	**0.3**	**-**	**34**	**28.6**	**17.3**	**0**	**-**	**78.7**
**Hispanic/Latino (%) ^a^**	**70.8**	**16**	**21.1**	**-**	**95.1**	**31.2**	**24.3**	**0**	**-**	**100**
**Concentrated disadvantage (yes/no) ^a^**	**84.2%**	**36.6%**	**0.0%**	**-**	**100.0%**	**27.0%**	**44.0%**	**0.0%**	**-**	**100.0%**
**Arizona health score ^c^**	**−3.2**	**1.7**	**−7.9**	**-**	**1.6**	**0**	**2.2**	**−7.9**	**-**	**4.8**
**Built Environment**										
**Streetlight brightness (unit: nW/cm^2^ sr^−1^** **) ^c^**	**40.1**	**14.9**	**4.2**	**-**	**91.5**	**66.3**	**20.7**	**0.2**	**-**	**100**
**Traffic (proximity/volume)^c^**	**13.3**	**13.1**	**0.1**	**-**	**88.9**	**51.1**	**31.2**	**0.1**	**-**	**99.9**
Parks (% total area) ^c^	47.9	20.2	12.2	**-**	97	53.9	24.1	12.2	-	100
Walkability (percentile) ^c^	59.2	21.2	6.7	**-**	99	60.8	24.2	1.3	-	100
Lack of supermarkets (%) ^c^	66.3	40.1	0.1	**-**	100	66.4	34.4	0.1	-	100
Full-service, served seated restaurants per square mile (#) ^d^	6	4.6	0	**-**	19.9	6.5	8	0	-	113.1
Fast food restaurants per square mile (count) ^d^	2	2.1	0	**-**	9.2	2.6	3.5	0	-	47.6
Alcoholic drinking places per square mile (e.g., bars; count) ^d^	1.4	2	0	**-**	8	1.2	2.6	0	-	45.3
Snack shops (e.g., coffee shops; count) ^d^	0.5	1.1	0	**-**	5.9	0.8	1.4	0	-	15.9

Data Sources: ^a^ American Community Survey, ^b^ The United States Department of Agriculture Food Access Research Atlas, ^c^ The Arizona Healthy Community Map, ^d^ The National Neighborhood Data Archive. **Bold** indicates a statistically significant (*p* < 0.05) *t*-test or proportion *t*-test between Sample tracts and Phoenix Chandler tracts.

**Table 2:** Coefficients from regression models predicting 2-h glucose; bivariate associations and associations net individual/household covariates Latino youth (*n* = 133), 2013–2015.

	Models 1						Models 2					
	B		95% CI			R^2^	B		95% CI			R^2^
**Neighborhood-Level Characteristics**												
**Neighborhood Housing/Transportation**												
Average Household Size (#)	−5.97	+	−13.17	-	1.23	0.02	−7.36	+	−14.88	-	0.16	0.07
Living in Same House 1 year (%)	−0.65	*	−1.22	-	−0.07	0.04	−0.83	**	−1.46	-	−0.19	0.11
Living in Same Neighborhood 7 years (%)	−0.17		−0.40	-	0.07	0.02	−0.23	+	−0.48	-	0.02	0.08
No Vehicle (%)	0.38	+	−0.003	-	0.08	0.03	0.50	*	0.09	-	0.91	0.09
Rental vacancy (%)	0.66	*	0.11	-	1.21	0.03	0.81	**	0.26	-	1.37	0.09
**Neighborhood Sociodemographic**												
Gross Median Rent ($) in $100	−1.95	+	−4.09	-	0.19	0.03	−2.65	*	−4.87	-	−0.43	0.09
Low Income Tract (% Yes)	12.97		−5.34	-	31.29	0.02	14.47		−3.66	-	32.60	0.07
Poverty Rate (%)	0.15		−0.17	-	0.48	0.01	0.20		−0.15	-	0.55	0.06
Unemployment (%)	−0.10		−0.28	-	0.08	0.01	−0.12		−0.31	-	0.07	0.06
College graduates (%)	0.25		−0.40	-	0.89	0.01	0.27		−0.38	-	0.91	0.05
Hispanic/Latino (%)	−0.15		−0.48	-	0.17	0.01	−0.16		−0.49	-	0.18	0.06
Concentrated disadvantage (yes/no)	8.15		−3.84	-	20.15	0.01	8.82		−3.93	-	21.58	0.06
Arizona health score (#)	−0.87		−4.00	-	2.27	0.00	−1.01		−4.12	-	2.11	0.05
**Built and Food Environment**												
Streetlight brightness percentile (unit: nW/cm^2^ sr^−1^)	0.26	+	−0.03	-	0.55	0.02	0.32	*	0.02	-	0.63	0.08
Traffic (proximity/volume percentile)	−0.38	**	−0.63	-	−0.13	0.04	−0.43	***	−0.69	-	−0.16	0.09
Parks (% total area)	−0.04		−0.26	-	0.18	0.00	−0.02		−0.24	-	0.20	0.05
Walkability (score, percentile)	0.04		−0.16	-	0.24	0.00	0.08		−0.13	-	0.30	0.05
Lack of supermarkets (percentile)	0.001		−0.11	-	0.11	0.00	−0.01		−0.13	-	0.12	0.05
Full-service, served seated restaurants per square mile (count)	0.13		−0.66	-	0.93	0.00	0.41		−0.43	-	1.25	0.05
Fast food restaurants per square mile (count)	2.42	**	0.49	-	4.34	0.04	3.03	**	0.85	-	5.22	0.10
Alcoholic drinking places per square mile (e.g., bars; count)	0.19		−2.43		2.82	0.00	0.47		−2.50	-	3.45	0.05
Snack shops (e.g., coffee shops; #)	−3.25	*	−6.40		−0.09	0.02	−2.81		−6.24	-	0.61	0.06

*** *p* < 0.001, ** *p* < 0.01, * *p* < 0.05, + *p* < 0.10. Notes: Model 1 only includes the neighborhood variable predicting 2-h glucose. Model 2 includes each neighborhood covariate in a different model and adjusts for individual/household controls: respondent gender and age, household income, parental education, household income, and household size. Standard errors account for clustering.

**Table 3:** Coefficients from ordinary least squares regression models predicting 2-h glucose; joint neighborhood associations among Latino youth (*n* = 133), 2013–2015.

	Models 3						Model 4					
	B		95% CI			R^2^	B		95% CI			R^2^
**Neighborhood-Level Characteristics**												
**Neighborhood Housing/Transportation**												
Average Household Size (#)	−1.06		−9.55	-	7.43	0.14	−4.60		−19.43	-	10.22	0.30
Living in Same House 1 year (%)	−0.58	+	−1.28	-	0.11		−0.52		−1.17	-	0.12	
Living in Same Neighborhood 7 years (%)	−0.03		−0.31	-	0.24		0.05		−0.25	-	0.35	
No Vehicle (%)	0.21		−0.21	-	0.63		0.05		−0.80	-	0.91	
Rental vacancy (%)	0.44		−0.25	-	1.12		0.51		−0.30	-	1.32	
**Neighborhood Sociodemographic**												
Gross Median Rent ($) in $100	−1.77		−4.85	-	1.31	0.14	−0.86		−4.69	-	2.98	
Low Income Tract (% Yes)	8.14		−14.75	-	31.03		3.46		−24.73	-	31.65	
Poverty Rate (%)	−0.02		−0.49	-	0.45		−0.09		−0.69	-	0.51	
Unemployment (%)	−0.10		−0.31	-	0.11		−0.17		−0.40	-	0.07	
College graduates (%)	0.57		−0.49	-	1.63		0.07		−1.02	-	1.15	
Hispanic/Latino (%)	−0.14		−0.68	-	0.41		−0.15		−0.74	-	0.44	
Concentrated disadvantage (yes/no)	6.57		−7.87	-	21.02		15.91	+	−0.07	-	31.89	
Arizona health score (#)	−1.20		−6.49	-	4.09		2.81		−2.21	-	7.84	
**Built and Food Environment**												
Streetlight brightness (unit: nW/cm^2^ sr^−1^)	0.11		−0.27	-	0.50	0.16	−0.16		−0.69	-	0.36	
Traffic (proximity/volume)	−0.32	*	−0.62	-	−0.02		−0.43		−0.96	-	0.10	
Parks (% total area)	−0.02		−0.26	-	0.22		−0.25	+	−0.53	-	0.02	
Walkability (score, percentile)	0.08		−0.17	-	0.33		−0.03		−0.33	-	0.27	
Lack of supermarkets (%)	−0.04		−0.17	-	0.09		−0.02		−0.16	-	0.12	
Full-service, served seated restaurants per square mile (count)	−0.68		−1.96	-	0.59		−2.00	**	−3.51	-	−0.50	
Fast food restaurants per square mile (count)	3.34	*	0.50	-	6.19		5.48	***	2.52	-	8.45	
Alcoholic drinking places per square mile (e.g., bars; count)	−0.47		−2.97	-	2.03		−0.75		−3.51	-	2.00	
Snack shops (e.g., coffee shops; count)	−2.10		−5.37	-	1.09		−1.17		−4.74	-	2.41	

*** *p* < 0.001, ** *p* < 0.01, * *p* < 0.05, + *p* < 0.10. Notes: All models account for individual/household controls: respondent gender and age, household income, parental education, household income, and household size. Model 3 includes models in the different groups (i.e., housing/transportation in one model, sociodemographics in one model, and built and food environment in one model. Model 4 includes all neighborhood covariates in one model. Standard errors account for clustering.

## Data Availability

Given that the data focus on a pediatric population and include addresses they are private. Code is available upon request.

## Supplementary Materials

The supporting information can be downloaded at: https://www.mdpi.com/article/10.3390/ijerph19137920/s1.
