# Supplementary material for: Neighborhood Conditions and Type 2 Diabetes Risk among Latino Adolescents with Obesity in Phoenix
Source: Int J Environ Res Public Health. 2022 Jun 28;19(13):7920. doi: 10.3390/ijerph19137920 (PMC9265310; doi:10.3390/ijerph19137920)
Supplement: Supplementary file 1 [file ijerph-19-07920-s001.zip › ijerph-1766735-supplementary.pdf]

Supplemental Materials: Neighborhood Conditions and Type 2 Diabetes Risk among Obese Latino Adolescents in Phoenix

American Community Survey

Data on neighborhood housing, socioeconomic status, and demographics characteristics were gathered from the United States Census Bureau's American Community Survey (ACS). The ACS is an ongoing survey issued to subsets of the US population and collects more detailed information than the decennial census. While the ACS provides 1- and 5-year period estimates, we used the 5 – year estimates as the 1-year estimates were unavailable for many of our measures given the small sampling unit (i.e., tracts). Data from ACS were gathered and matched to the participant's baseline enrollment year in the study (i.e., 2013, 2014, or 2015).

The United States Department of Agriculture Food Access Research Atlas

The United States Department of Agriculture Food Access Research Atlas is a collection of food access and economic indicators. The Food Access Research Atlas was developed from a 2008 bill that aimed to better understand the effects of living in low-income areas and low-access to food stores ("food deserts"). These indicators include distance to the nearest super market, vehicle availability, household income, and poverty. Data from 2015 were available and used from the Food Access Research Atlas, as this best coincided with the majority of the participants' baseline enrollment year.

The Arizona Healthy Community Map

The Arizona Healthy Community Map is a collection of 36 social and environmental determinants of health used to measure neighborhood health in Arizona, and is the result of a joint project between the Arizona State University School of Geographical Sciences and Urban Planning and Vitalyst Health Foundation (for more information please see: https://sgsup.asu.edu/sparc/azhealthmap). These indicators were developed based on the current

literature on neighborhood health, previously developed healthy community maps, and advisory input from key health – related state stakeholders. The Arizona Healthy Community map contains information on the domains of: access to care; affordable quality housing; community safety; economic opportunity; educational opportunity; environmental quality; food access; health community design; parks and recreation; social and cultural cohesion; social justice; and transportation. A composite index of all variables measuring neighborhood health was available and also used in the analysis. Only data from 2017 were available and used from the Arizona Health Community Map.

The National Neighborhood Data Archive

The National Neighborhood Data Archive (NaNDA): Eating and Drinking Places by Census Tract, United States, 2003-2017 was developed by researchers at the University of Michigan Institute for Social Research, and is a collection of indicators on the number and density of eating places in the United States. University of Michigan researchers compiled these indicators using establishment data from the National Establishment Time Series database and Census data from the United States Census Bureau; Neighborhood Socioeconomic and Demographic Characteristics of Census Tracts, United States, 2000-2010; and the Socioeconomic Status and Demographic Characteristics of Census Tracts, United States, 2008-2017. These indicators, available at the census tract level, include: full-service restaurants; fast-food restaurants; alcoholic drinking places; and snack shops. Data from 2014 were used, as this was the midpoint for all participants' baseline enrollment year.
